# Supplementary material for: SyNDI: synchronous network data integration framework
Source: BMC Bioinformatics. 2018 Nov 6;19:403. doi: 10.1186/s12859-018-2426-5 (PMC6219086; doi:10.1186/s12859-018-2426-5)
Supplement: Supplementary file 5 — Files of the example presented in Additional file 4. This additional file is a zip package that contains all files used in Additional file 4. Detailed descriptions of each of these files are presented in a README file included in this zip package. (GZ 30411 kb) [file 12859_2018_2426_MOESM5_ESM.gz › SyNDITools/Meme2Fimo/meme_4.9.0_4.tar.gz!/meme_4.9.0/website/cgi-bin/meme-chip.tmpl]

MEME-ChIP - Submission form


Select a file of FASTA formatted
DNA sequences or paste in actual FASTA formatted DNA sequences to find motifs.

See the sample sequences
to get an idea of input that works well for MEME-ChIP.

[ close ]

MEME-ChIP will compare any found motifs to this set and also check if
any are centrally enriched.

MEME-ChIP can use databases installed on the server or search a user
uploaded database in MEME DNA motif
format by selecting the "Upload Your Own Database" option. For more
information browse the
supported databases.

[ close ]

An uploaded database is a normal MEME DNA motif file with lots of motifs. The uploaded database must
be smaller than 5MB.

[ close ]

The email is used to notify you of your results.

[ close ]

The job description will be included in the notification email you
receive and in the MEME-ChIP output.

[ close ]

When using the RNA sequences you should select this option to ensure
that only the given strand is scanned for motifs.

[ close ]

By default MEME-ChIP will determine the background Markov model from
the provided sequences. Alternately a file containing a background model may be input here.

The downloadable version of the MEME Suite contains a script named
"fasta-get-markov" that you can use to create background model files in
the correct format from FASTA sequence files.

[ close ]

This is where you tell MEME how you believe occurrences of the motifs
are distributed among the sequences. Selecting the correct type of
distribution improves the sensitivity and quality of the motif search.

Zero or one occurrence per sequence
:   MEME assumes that each sequence may contain **at most** one
    occurrence of each motif. This option is useful when you suspect that
    some motifs may be missing from some of the sequences. In that case, the
    motifs found will be more accurate than using the one occurrence per
    sequence option. This option takes more computer time than the one
    occurrence per sequence option (about twice as much) and is slightly less
    sensitive to weak motifs present in all of the sequences.

One occurrence per sequence
:   MEME assumes that each sequence in the dataset contains
    **exactly** one occurrence of each motif. This option is the fastest
    and most sensitive but the motifs returned by MEME may be "blurry" if
    any of the sequences is missing them.

Any number of repetitions
:   MEME assumes each sequence may contain **any number** of
    non-overlapping occurrences of each motif. This option is useful when
    you suspect that motifs repeat multiple times within a single sequence.
    In that case, the motifs found will be much more accurate than using one
    of the other options. This option can also be used to discover repeats
    within a single sequence. This option takes much more computer time than
    the one occurrence per sequence option (about ten times as much) and is
    somewhat less sensitive to weak motifs which do not repeat within a
    single sequence than the other two options.

[ close ]

MEME will keep searching until it finds this many motifs or it hits
some other threshold like the maximum run time. Note that MEME does not
use an E-value threhold like DREME so you should always check the E-value
of any found motifs.

[ close ]

This is the width (number of characters in the sequence pattern) of a
single motif. MEME chooses the optimal width of each motif individually
using a statistical heuristic function. You can choose different limits
for the minimum and maximum motif widths that MEME will consider. The
width of each motif that MEME reports will lie within the limits you
choose.

[ close ]

This is the total number of sites in the training set where a single
motif occurs. You can choose different limits for the minimum and maximum
number of occurrences that MEME will consider. If you have prior knowledge
about the number of occurrences that motifs have in your training set,
limiting MEME's search in this way can can increase the likelihood of
MEME finding true motifs.

MEME chooses the number of occurrences to report for each motif by
optimizing a statistical heuristic function, restricting the number of
occurrences to the range you give here, or using defaults described below
if you leave these fields deselected.

| Distribution | Minimum | Maximum |
| --- | --- | --- |
| Zero or one occurrence per sequence | sqrt(n) | n |
| One occurrence per sequence | n | n |
| Any number of repetitions | sqrt(n) | min(5\*n, 600) |

[ close ]

Checking this box causes MEME to search only for DNA palindromes. This
causes MEME to average the letter frequencies in corresponding motif
columns together. For instance, if the width of the motif is 10, columns
1 and 10, 2 and 9, 3 and 8, etc., are averaged together. The averaging
combines the frequency of A in one column with T in the other, and the
frequency of C in one column with G in the other. If this box is not
checked, the columns are not averaged together.

[ close ]

DREME will keep finding motifs until it hits one of the limits.

[ close ]

Specify a minimum score for a match to be considered. If a sequence does
not have any matches which meet this minimum score for a given motif, then
that sequence will not be considered for that motif.

[ close ]

This option limits the maximum region size that CentriMo will test.
This option is useful if you have sequences longer than the normal 500bp
and can help cut-down the multiple testing correction. When this option is
not supplied CentriMo will test region sizes up to one less than the maximum
number of places that a given motif can align to the sequence.

[ close ]

This is the E-value threshold CentriMo uses for reporting enriched
central regions for motifs. If multiple enriched regions overlap then the region
with the best *p*-value and smallest size will be output.

[ close ]

This option causes all regions up to the maximum region size to be
considered even if they are not in the center. This can be useful with
non-symetric data.

[ close ]

This option enables you to store the sequence identifies which have
their best match in the best region for each motif. This option can
potentially make the CentriMo output file size much larger.

[ close ]

|  |  |
| --- | --- |
|  | MEME Suite  The MEME Suite web application requires the use of JavaScript  Javascript doesn't seem to be available on your browser.  Version  Use this form to submit DNA sequences to MEME-ChIP. MEME-ChIP is designed especially for discovering motifs in **LARGE** (50MB maximum) sets of short (around 500bp) DNA sequences centered on locations of interest such as those produced by ChIP-seq experiments.  Data Submission Form Perform motif discovery and enrichment on large DNA datasets.  Input the sequences  Enter DNA sequences in which you want to find motifs  or paste the sequences   Input the motif database  Upload Your Own Database  Input motifs to upload  Select a MEME motif database file.   Input job queue details  Enter your email address.  Re-enter your email address.     Optionally enter a job description.   ▶ ▼  Universal options hidden modifications! [Reset]  Scan both DNA strands?  scan given strand only  Use a custom background? Custom background:   ▶ ▼  MEME options hidden modifications! [Reset]  What is the expected motif site distribution?  **Zero or one** occurrence per sequence **One** occurrence per sequence **Any number** of repetitions   How many motifs should MEME find? Count of motifs:   What width motifs should MEME find?  Minimum width:  Maximum width:   How many sites per motif is acceptable?   Minimum sites:   Maximum sites:  Should MEME restrict the search to palindromes?  look for palindromes only   ▶ ▼  DREME options hidden modifications! [Reset]  How should DREME limit its search?  *E*-value ≤   Count ≤   ▶ ▼  CentriMo options hidden modifications! [Reset]  Set a minimum acceptable match score (bits) score ≥   Set the maximum allowed width of central region  region width ≤   Set *E*-value threshold for reporting centrally enriched regions *E*-value ≤   Find uncentered regions  Run CentriMo in local mode to find uncentered regions  Include sequence IDs  Include a list of matching sequence ids |
